# Rhombohedral/Cubic In_2_O_3_ Phase Junction Hybridized with Polymeric Carbon Nitride for Photodegradation of Organic Pollutants

**DOI:** 10.3390/ijms232214293

**Published:** 2022-11-18

**Authors:** Xiaorong Cai, Yaning Wang, Shuting Tang, Liuye Mo, Zhe Leng, Yixian Zang, Fei Jing, Shaohong Zang

**Affiliations:** 1 Institute of Innovation & Application, National Engineering Research Center For Marine Aquaculture, Zhejiang Ocean University, Zhoushan 316022, China; 2 Donghai Laboratory, Zhoushan 316021, China

**Keywords:** photocatalysis, photodegradation, carbon nitride, phase junction, MOF

## Abstract

In recent studies, phase junctions constructed as photocatalysts have been found to possess great prospects for organic degradation with visible light. In this study, we designed an elaborate rhombohedral corundum/cubic In_2_O_3_ phase junction (named MIO) combined with polymeric carbon nitride (PCN) via an in situ calcination method. The performance of the MIO/PCN composites was measured by photodegradation of Rhodamine B under LED light (λ = 420 nm) irradiation. The excellent performance of MIO/PCN could be attributed to the intimate interface contact between MIO and PCN, which provides a reliable charge transmission channel, thereby improving the separation efficiency of charge carriers. Photocatalytic degradation experiments with different quenchers were also executed. The results suggest that the superoxide anion radicals (O_2_^−^) and hydroxyl radicals (·OH) played the main roles in the reaction, as opposed to the other scavengers. Moreover, the stability of the MIO/PCN composites was particularly good in the four cycling photocatalytic reactions. This work illustrates that MOF-modified materials have great potential for solving environmental pollution without creating secondary pollution.

## 1. Introduction

Organic dye pollutants in wastewater have constantly been a concern with the development of society [1,2,3]. Their toxicity and carcinogenicity always threaten ecological balance and biological health. The processing methods of organic dyes generally include adsorption, physical/chemical precipitation, biological methods, and photodegradation. Among them, solar-driven degradation by semiconductor photocatalysts has great potential in resolving organic dye pollution due to its convenience, eco-friendliness, and low cost [4,5]. It is well known that the most important part of photocatalysis is the catalysts because they accelerate the reaction process and improve the degradation efficiency in organic dye degradation reactions [6,7]. Therefore, photocatalysts working under visible light irradiation with outstanding photodegradation efficiency still need further exploration.

As an organic representative, polymeric carbon nitride (PCN) is a star material in photocatalysis owing to its suitable energy band position, excellent stability, and simple synthetic applications [8,9,10,11]. The extended π-conjugated systems consisting by sp^2^-hybridized C and N atoms have been widely used for studies on energy and the environment [12]. The suitable bandgap of PCN (2.7 eV) enables it to harvest visible light and surmount the endothermic character of water-splitting reactions (theoretically, 1.23 eV) [13]. However, as a non-metal photocatalyst, the insufficient capacity of charge carrier transfer results in the unsatisfactory photocatalytic ability of PCN [12,14]. Many strategies have been used to remedy this issue, such as morphological control, element doping, cocatalyst loading, band structure engineering, and heterojunction construction [15,16,17,18]. Among various methods, heterojunction construction has been proven to be an easy yet effective method to accelerate the migration of charge carriers [19,20,21]. Therefore, coupling with proper semiconductors can also improve the photoactivity of PCN.

Previous reports revealed that the phase junction of polymorph semiconductors plays a vital role in charge separation [22,23]. Photo-induced charge transfer between two phases is driven by the built-in electric field in the phase junction, resulting in enhanced photocatalysis. Taking TiO_2_ as an example, Li’s group established that the photocatalytic activity is directly influenced by the surface phase structure [24,25]. And the phase junction consisting of anatase and rutile particles performed better in photocatalytic H_2_ evolution. Hao et al. fabricated a novel CdS phase junction with bonding region-width-control and resolved photocorrosion and phase exclusion of CdS [26]. The best performance reached as much as 60-fold that of the single cubic or hexagonal phase. Liu et al. reported a black/red phase junction phosphorus with faster charge transport properties benefiting from the appropriate band structures [27]. The theoretical and experimental data have indicated that different kinds of phase junction materials stand out in photo-to-electron conversion efficiency. 

Recently, a metal organic framework (MOF)-derived rhombohedral/cubic In_2_O_3_ phase junction (named MIO) was reported for solar-driven water splitting [28]. Because it has no cytotoxicity or cellular ROS generation, and is easy to obtain, MIO has exhibited a growth potential in photocatalysis, such as H_2_ production, CO_2_ reduction, and pollutant degradation [29]. Theoretical calculations have illustrated that the photo-generated electrons transfer from c-In_2_O_3_ to rh-In_2_O_3_ was efficient in preventing the recombination of charge carriers. Previous research has mainly focused on cubic In_2_O_3_-based semiconductor (c-In_2_O_3_), but rarely on its phase junction [30,31]. For example, Wang et al. designed ZnIn_2_S_4_-In_2_O_3_ nanotubes with good stability for CO_2_ reduction [32]. Li’s group reported core-shell In_2_O_3_@Carbon nanoparticles for photocatalytic hydrogen evolution [33]. The improved accessibility between c-In_2_O_3_ and carbon nanoparticles not only favored the efficient separation of charge carriers, but also enhanced the optical absorption. Sun et al. synthesized G-C_3_N_4_/In_2_O_3_ composites for effective formaldehyde detection [34]. Xu et al. reported a carbon-doped In_2_O_3_/g-C_3_N_4_ heterojunction for photoreduction of CO_2_ [35]. Jin and Uddin et al. reported c-In_2_O_3_ hybridization with boron-doped and oxygen-doped carbon nitride for photodegradation, respectively [36,37]. Both of them showed a superior kinetic degradation rate rather than either In_2_O_3_ or PCN alone. Although the investigation of cubic In_2_O_3_-heterojunction in photocatalysis has made some progress, the In_2_O_3_ phase junction has not been looked at in detail.

**Scheme 1:**
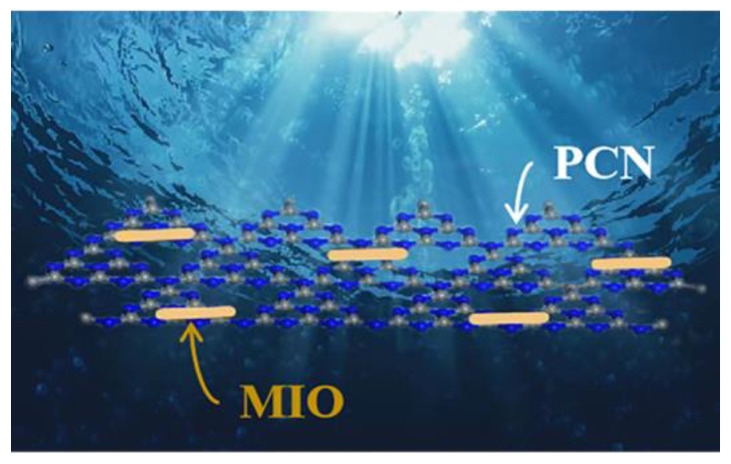
Diagram of the MIO/PCN sample.

In this study, we designed a MOF-derived phase junction In_2_O_3_/PCN (named MIO/PCN, Scheme 1) heterojunction prepared by an in situ method in which the two precursors of MIO and PCN were mixed and then calcined at 500 °C. The MIO/PCN heterojunctions exhibited better visible light absorption, more active sites, and faster charge transfer. The optimal photodegradation activity of MB by MIO/PCN was about 95- and 19-fold that of the MIO and PCN, respectively. In addition, the MIO/PCN composites exhibited excellent stability after four-cycle photodegradation. The main active species were determined to be superoxide anion radicals (O_2_^−^) and hydroxyl radicals (·OH) by adding scavengers to the degradation reaction, and were further confirmed by EPR analysis. Based on UV-Vis DRS, PL/TRPL, and photoelectrochemical tests, the possible photodegradation reaction mechanism of MIO/PCN is discussed. This present strategy may promote new ideas for exploring the application field of the phase junction oxides and other related materials.

## 2. Results and Discussion

**Figure 1:**
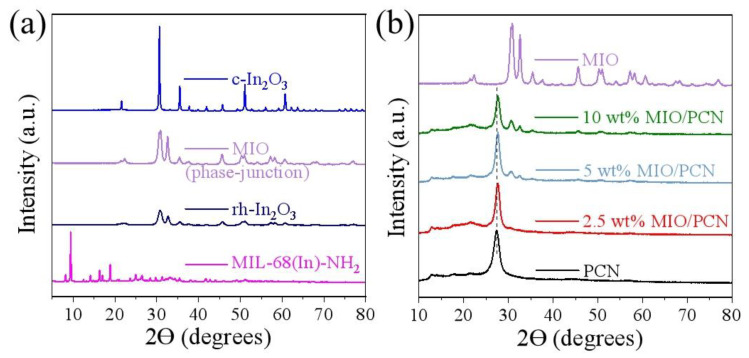
XRD spectra of (**a**) MIL-68(In)-NH_2_ and different phases of In_2_O_3_, and (**b**) pure PCN, MIO, and x wt% MIO/PCN composites.

The crystal structure of x wt% MIO/PCN composites was confirmed by X-ray diffraction (XRD) analysis. As observed from the XRD pattern in Figure 1a, the crystal structure of the MIL-68(In)-NH_2_ precursor and MIO were in accordance with the previous report and no other characteristic peak was detected, respectively [31]. MIO calcined at 500 °C is composed of the mixed phase of rh-In_2_O_3_ (PDF No. 22-0336) and c-In_2_O_3_ (PDF No. 06-0416) [28]. And no other peaks appeared, indicating good purity of the In_2_O_3_ phase-junction. In Figure 1b, there were two characteristic diffraction peaks of the pure PCN sample located at 2θ = 12.8° and 27.3°, which corresponded to the (100) and (002) planes, respectively (JCPDS card No. 87-1526). The former was assigned to the packing motif of heptazine units in plane and the latter originated from the stacking of the conjugated aromatic system in the interlayer [18]. In the XRD patterns of MIO/PCN composites, the peak intensity of MIO became more muscular while the content of MIO increased. It was identified that MIO and PCN existed in the MIO/PCN composites. In addition, the peaks of the pure PCN sample located at 2θ = 27.3° moved to high degree, indicating that the spacing d was reduced according to the Bragg equation. That is because of the interaction between PCN and MIO and is beneficial to charge transfer [8]. 

**Figure 2:**
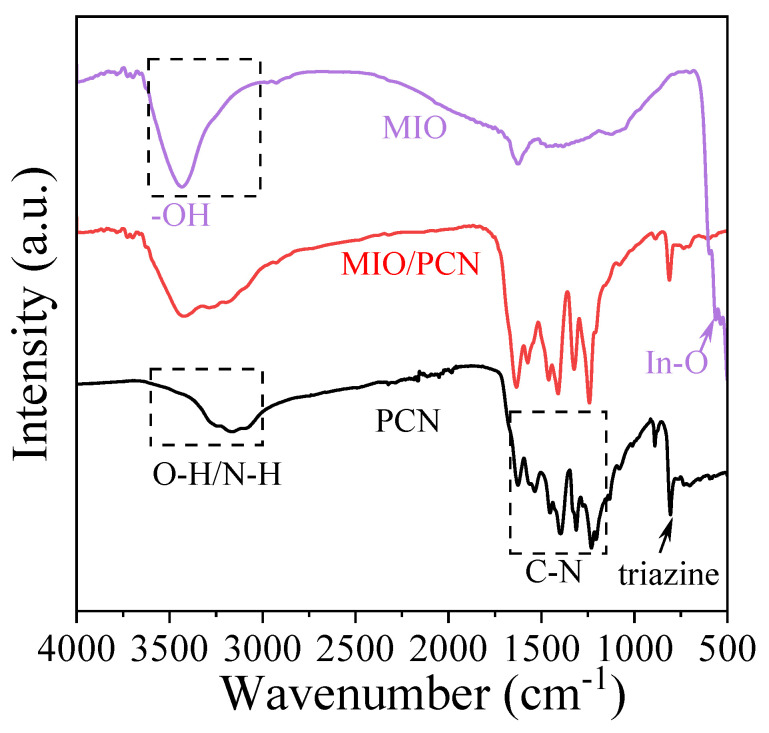
FT-IR spectra of pure PCN, MIO, and 2.5 wt% MIO/PCN composites.

The FT-IR spectra of PCN, MIO, and MIO/PCN are shown in Figure 2 to illustrate the information pertaining to functional groups. In the FT-IR spectra of PCN, the broad peaks located at 3000–3600 cm^−1^ indicate the stretching vibration of O-H, N-H, and hydrogen-bonding interactions [38,39]. The characteristic peaks at 1200–1600 cm^−1^ correspond to the C-N skeleton of PCN. The breathing mode of the triazine units appeared at 800 cm^−1^, meaning the presence of -NH and -NH_2_ groups [39]. In that of MIO, the characteristic peaks at 570 cm^−1^ were determined to be In-O asymmetric stretching, and the peaks at 3000–3400 cm^−1^ correspond to the stretching vibration of the -OH hydrogen bond [11,34]. As shown in the FT-IR spectra of MIO/PCN, all the characteristic peaks of PCN appeared, indicating the main structure of PCN was not changed. Although the In-O peak was not observed, possibly because of the small amount of MIO in composites and the weak peak intensity, the characteristic peaks of PCN and MIO appeared simultaneously at 3000–3600 cm^−1^. The FT-IR spectra further confirmed the formation of MIO/PCN.

**Figure 3:**
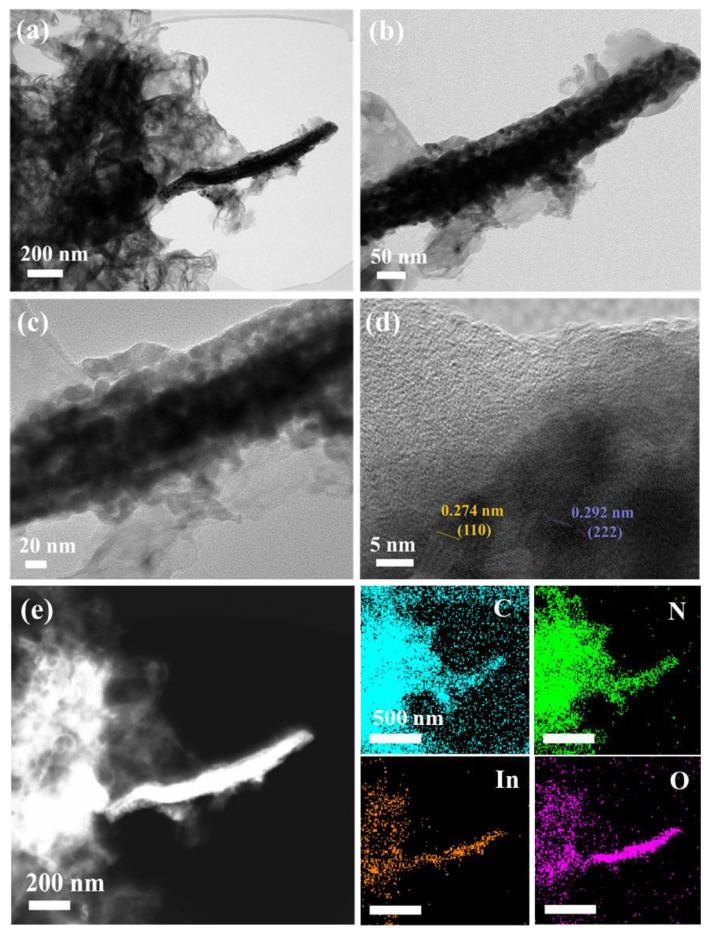
(**a**–**d**) TEM images, (**e**) EDX mapping of 2.5 wt% MIO/PCN composites.

SEM and TEM images shown in Figure 3 and Figure S1 are to illustrate the morphology of the composites. In Figure S1, the shape of MIL-68(In)-NH_2_ is a rod with a smooth surface and that of PCN is a nanosheet, which was in accordance with the previous reports. After in situ calcination loading, the morphology of MIO/PCN had no noticeable change compared with pure PCN, whereas MIO could not be observed. In the TEM images of MIO/PCN (Figure 3a–c), the rod-like MIO was surrounded by nanosheets of carbon nitride. This was mainly due to the in situ method, which mixed the two precursors of carbon nitride and indium oxide first, which were then calcined together. Moreover, that is why only PCN could be observed in the SEM images of MIO/PCN. In Figure 3d, the HRTEM image of MIO/PCN, two lattice fringes could be observed in MIO/PCN with layer distances of 0.274 nm and 0.292 nm, corresponding to (110) rh-In_2_O_3_ crystal planes and (222) c-In_2_O_3_ crystal planes, respectively. In addition, amorphous PCN nanosheets were observed. The EDX mapping in Figure 3e showed that C, N, In, and O were mainly distributed across the whole nanocomposites. This phenomenon may be caused by the in situ method. The analysis of XRD, FT-IR spectra, SEM, and TEM images confirmed the formation of MIO/PCN composites.

**Figure 4:**
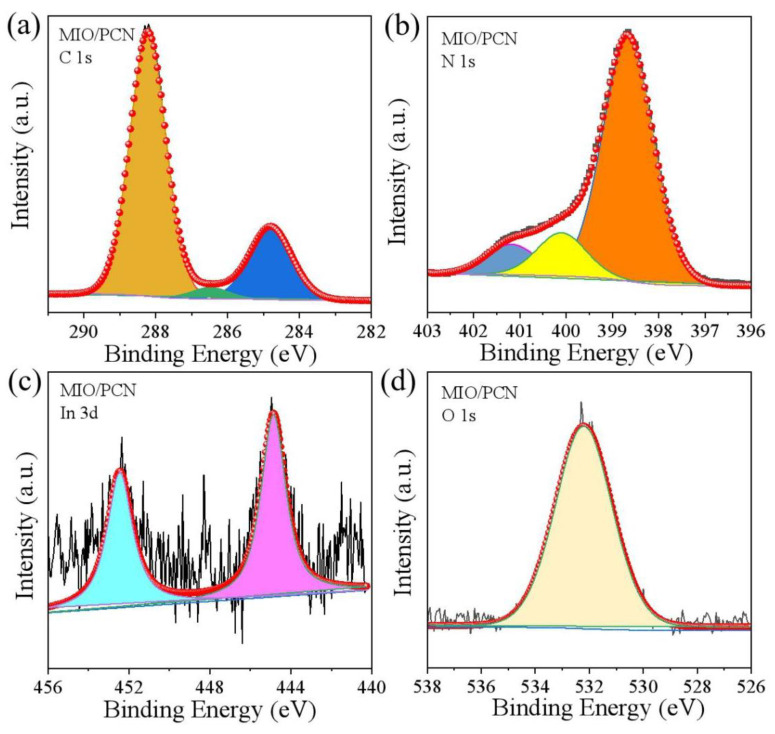
XPS spectra of 2.5 wt% MIO/PCN (**a**) C 1s, (**b**) N 1s, (**c**) In 3d, (**d**) O 1s.

To analyze the chemical components and chemical states in 2.5 wt% MIO/PCN, XPS research was then conducted. Consistent with the EDX results, In, O, C, and N elements were all detected in the XPS survey spectra (Figure S2), which further proves the co-existence of MIO and PCN in the composites. High-resolution XPS spectrum of the elements in MIO/PCN was also carried out. In Figure 4a, there were three peaks of C 1s at the binding energy of 288.2, 286.4, and 284.8 eV. The first peak was vested in N-C=N of the triazine ring, the second peak was attributed to C-O=C bond, while the last ones were vested in the C=C group of PCN [17,38]. In Figure 4b, the peaks of N 1s were divided into three characteristic peaks. The characteristic peak at 401.0 eV was caused by uncondensed C-N-H groups on the surface of PCN [9,10]. The last two forms of N 1s, located at 400.1 and 398.6 eV, belonged to the tertiary N-(C)_3_ groups and sp^2^-hybridized nitrogen (C=N-C) in aromatic triazine rings, respectively [39]. Both of them and sp^2^-C constituted the heptazine C_6_N_7_ units of PCN [40]. In Figure 4c, the two XPS peaks located at 444.8 and 452.5 eV corresponded to the spin-orbit coupling of In 3d_5/2_ and In 3d_3/2_ of MIO [41]. The peak in Figure 4d at 532.0 eV belongs to O_C_ orbitals fitted to chemisorbed oxygen species. The XPS analysis indicated the co-existence of MIO and PCN in the composites.

**Figure 5:**
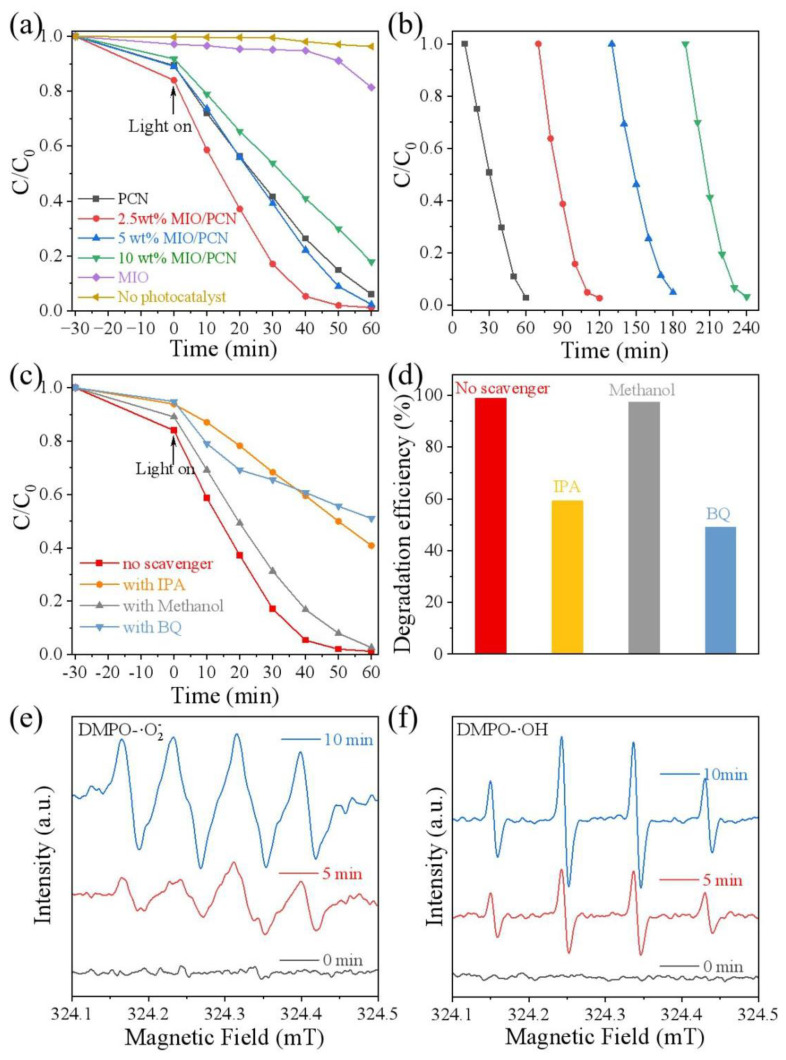
Photodegradation of RhB under LED light irradiation (λ = 420 nm) (**a**) by x wt% MIO/PCN samples, (**b**) four cycle tests by 2.5 wt% MIO/PCN, (**c**,**d**) different scavengers on the degradation of RhB of 2.5 wt% MIO/PCN, EPR spectra of 2.5 wt% MIO/PCN for the detection of (**e**) DMPO-·O_2_^−^, (**f**) DMPO-·OH.

The performance of the MIO/PCN composites was measured by photodegradation RhB under LED light irradiation (λ = 420 nm). It can be seen in Figure 5a that the adsorption of RhB on pure PCN, MIO, or x wt% MIO/PCN was faint. In the absence of a catalyst, the photodegradation of RhB hardly occurred. This further illustrated the crucial importance of a catalyst in photodegradation of RhB. However, the photocatalytic activity of the pure MIO sample showed poor degradation capacity (17%) for RhB while that of PCN was 90% in 60 min. Experimental results indicated that controlling the ratio of MIO in the MIO/PCN sample was of great importance in achieving optimal photocatalytic degradation [42]. The 2.5 wt% MIO/PCN sample exhibited the best activity, reaching almost 100% degradation within 50 min under LED light irradiation (λ = 420 nm). The photodegradation efficiency of MIO/PCN samples was obviously enhanced compared to that of pure MIO. The photocatalytic stability of the 2.5 wt% MIO/PCN sample was tested by cycle degradation of RhB (Figure 5b). After four runs of continuous reaction, the 2.5 wt% MIO/PCN sample still exhibited stable photodegradation efficiency. The experimental results indicate that the MIO/PCN sample had good stability and the enhanced photodegradation activity of RhB was due to the introduction of MIO in the composites.

Photodegradation of RhB occurred because of the active species produced in MIO/PCN during the reaction. Therefore, the main active species were tested under the same photocatalytic degradation condition except for adding different scavengers. The scavengers included 1, 4-benzoquinone (BQ), methyl alcohol (MeOH), and isopropyl alcohol (IPA), which capture superoxide radicals (·O_2_^−^), holes (h^+^), and hydroxyl radicals (·OH), respectively [43]. Figure 5c,d shows that significantly decreased efficiency of the photodegradation occurred when BQ and IPA were added to the system. Additionally, there was no obvious change after the addition of MeOH in photocatalytic degradation. The photocatalytic experiment with scavengers suggested that ·O_2_^−^ and OH were the major active species in the photodegradation reaction of RhB [44].

The generation of the main active species on the MIO/PCN composite under visible-light irradiation was also probed by a 5,5-dimethyl-1-pyrroline N-oxide (DMPO) spin-trapping electron paramagnetic resonance (EPR) technique. In Figure 5e, when the light was off, no EPR signal appeared. When the light was on, a strong EPR signal of DMPO-·O_2_^−^ appeared. Additionally, when the light was kept on for another five minutes, the signals were measurably enhanced. As shown in Figure 5f, the characteristic signals of the DMPO-·OH radical emerged after visible light irradiation. It can be concluded that both ·O_2_^−^ and ·OH are the major reactive species in photodegradation of RhB by 2.5 wt% MIO/PCN photocatalyst. The EPR spectra is in accordance with the results of the capture experiments of active species [45]. By the above experimental studies, the possible mechanism was speculated as follows [46,47]:

MIO/PCN + *hv* → e^−^ + h^+^

e^−^ + O_2_ →·O_2_^−^

h^+^ + H_2_O/OH^−^ →·OH + H^+^

O_2_^−^, OH + RhB → products

**Figure 6:**
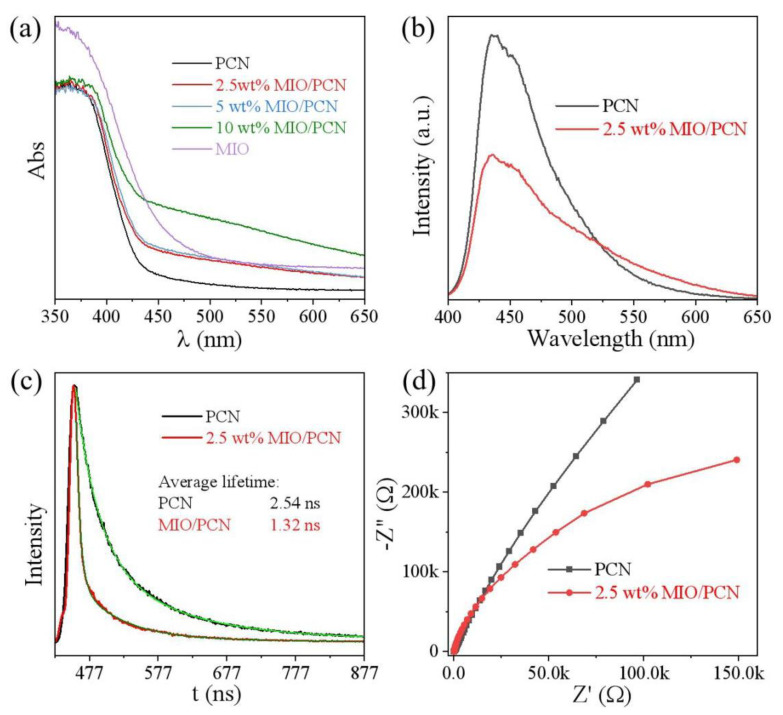
(**a**) UV/Vis DRS spectra, (**b**) room temperature PL spectra, (**c**) time-resolved PL spectra recorded at 298 K, (**d**) EIS Nyquist plots of pure PCN and MIO/PCN composites.

The optical and photoelectrical properties of MIO/PCN were investigated to explore the mechanism of enhanced photoactivity. In Figure 6a, there was a red shift of the DRS curve with increasing content of MIO in MIO/PCN. Broadened visible light absorption is vitally essential for increasing photocatalytic activity [48,49]. PL and TRPL spectra were used to explore the charge-carrier separation and migration behavior. It can be observed from Figure 6b that the fluorescence intensity of 2.5 wt% MIO/PCN was apparently quenched after being modified by MIO, indicating that the charge recombination was efficiently suppressed [17]. The average lifetime of PCN and 2.5 wt% MIO/PCN composite in Figure 6c was 2.54 ns and 1.32 ns. The rapid charge-carrier transfer from MIO to PCN resulted in a shorter PL lifetime [14,46]. Furthermore, the charge separation was further explored by the electrochemical impedance spectra (EIS). The evidently decreased Nyquist radius in Figure 6d illustrates that the resistance of 2.5 wt% MIO/PCN was much smaller than that of PCN [50,51]. The reduced resistance was conducive to the rapid migration of charge carriers. These results convincingly prove that the construction of MIO/PCN could promote charge separation and transfer.

**Figure 7:**
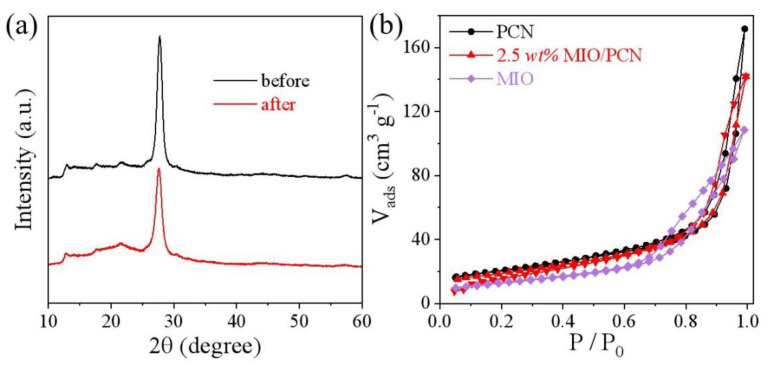
(**a**) XRD pattern of 2.5 wt% MIO/PCN sample before and after reaction, (**b**) low-temperature N_2_ adsorption-desorption isotherms of PCN, MIO, and 2.5 wt% MIO/PCN.

**Scheme 2:**
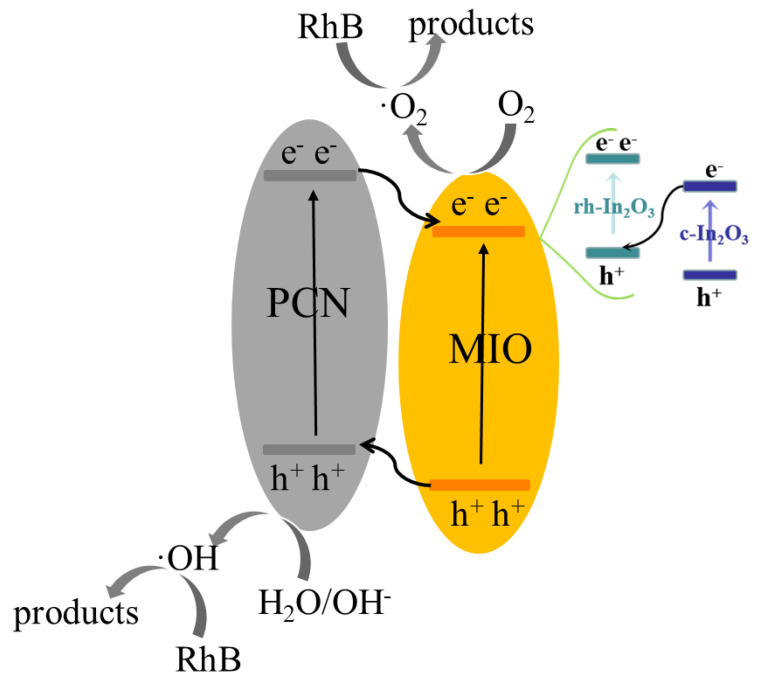
Possible reaction mechanism in photocatalytic reaction by MIO/PCN.

In order to exclude the influence of structural change of the photocatalyst, XRD analysis was conducted. As shown in Figure 7a, the structure of the MIO/PCN did not change before or after photodegradation of RhB. This indicates that the impact of structural changes of photocatalysts on their enhanced photoactivity can be excluded. In Figure 7b, the BET surface areas of PCN and MIO were determined as 72 and 45 m^2^g^−1^, respectively, while that of the MIO/PCN sample was 65 m^2^g^−1^. Compared with pure PCN, the slightly decreased specific surface areas of MIO/PCN was due to the addition of MIL-68(In)-NH_2_ by the in situ calcination [7,32]. The combination of the two block substances resulted in the decrease of BET-specific surface area. Therefore, the MIO/PCN composites performed well in photodegradation of RhB because of boosting solar absorption, fasting charge transfer, and being suppressed charge recombination. Based on the above discussion, a possible mechanism of charge transfer route in the MIO/PCN composites was proposed, as shown in Scheme 2. Irradiated by visible light, the electrons (e^−^) excited by light in the valence band (VB) of PCN transferred to the conduction band (CB) of PCN, and then flowed to the CB of MIO. Certainly, part of the electrons and holes recombined before the reaction. In addition, the electrons that migrated to the surface of photocatalysts reacted with O_2_ to form ·O_2_^−^. The holes in the CB reacted with H_2_O/OH^−^ to generate ·OH. The two active species, ·O_2_^−^/OH, attacked the RhB pollutants and generated harmless products, even including water and carbon dioxide.

## 3. Materials and Methods

Materials: All chemicals were used without further purification.

Indium nitrate hydrate (In(NO_3_)_3_·4H_2_O), 2-aminoterephthalic acid, N,N-dimethyl formamide (DMF), indium chloride tetrahydrate (InCl_3_·4H_2_O) and urea were purchased from Sinopharm Chemical Reagent Co., Ltd. (Shanghai, China). N,N-dimethylformamide (DMF) was purchased from Macklin Biochemical Co., Ltd. (Shanghai, China). All reagents were of analytical grade and were used without further purification.

### 3.1. Preparation of MIL-68(In)-NH_2_

The precursor MIL-68(In)-NH_2_ was prepared using a hydrothermal method [26]. Typically, a certain amount of 2-aminoterephthalic acid and In(NO_3_)_3_·xH_2_O were dissolved in DMF and the hydrothermal reaction occurred in an oven. After the reaction ended, the obtained products were washed with methanol and dried overnight at 80 °C.

### 3.2. Preparation of MIO/PCN and PCN

MIO/PCN samples were obtained using an in situ method [8]. Different amounts of MIL-68(In)-NH_2_ mixed with urea and calcined at 500 °C for 2 h in a muffle furnace. The products were collected and named x wt% MIO/PCN, where x means the calculated conversion rate of MIO.

Pure PCN was synthesized under the same conditions without the addition of MIL-68(In)-NH_2_.

### 3.3. Characterization 

XRD patterns were measured on an X-ray diffractometer (D/MAX-2200, Rigaku Company) to examine the crystal structure of samples. FT-IR spectra was used to analyze the functional groups of samples by using an ALPHA-P spectrometer. The morphology and elemental mapping images of samples were characterized by field-emission scanning electron microscopy (SEM, JSM-6700F) and transmission electron microscopy (TEM, JEOL JEM 2100F). The chemical valence of the elements in the samples was obtained using X-ray photoelectron spectroscopy (XPS, Thermo ESCALAB 250XI, standard peak is the C 1s peak at 284.8 eV). Nitrogen adsorption-desorption isotherms and the Brunauer-Emmett-Teller (BET) surface areas were collected at 77 K using Micromeritics ASAP2010 equipment. The visible-light absorption of samples was measured by UV-visible diffuse reflectance spectra (UV-DRS, Cary 500 Scan Spectrophotometer, Varian, Palo Alto, CA, USA). PL was performed on Varian Cary Eclipse (Agilent, Santa Clara, CA, USA) to research the recombination of charge carriers of samples. Electrochemical impedance spectroscopy (EIS) of samples was measured on an electrochemical workstation (Shanghai chenhua) in a standard three-electrode system in which the working electrode was the FTO glass with synthesized samples (10 mg catalyst in 1 mL 1% ethanol and 0.5 mL Nafion with an active area of 1 cm^2^); the reference electrode was the Ag/AgCl electrode; and the counter electrode was Pt wire. The electrolyte was 0.4 M Na_2_SO_4_ aqueous solution.

### 3.4. Photocatalytic Rhodamine b Degradation

The photocatalytic performance test was conducted under LED lamp (λ = 420 nm) irradiation. In the reaction, 10 mg samples were put into 50 mL 20 ppm rhodamine B (RhB) solution and stirred for 30 min under dark condition. Then the mixture was illuminated under a LED lamp (λ = 420 nm) for 1h. The original solution concentration was labeled C_0_. During the irradiation, the suspension (2–3 mL) was taken from the dispersion every 10 min, and the clarified reaction solution (concentration C) could be obtained by filtering with a needle filter. The absorbance of the solution at 664 nm was determined using a Shimazu UV-2600 UV-Vis spectrophotometer. The degradation efficiency of the RhB solution was calculated according to the formula D = ln (C/C_0_) × 100%. The absorption method replaced C_0_ and C according to the Lambert–Beer law.

## 4. Conclusions

In conclusion, the MIO/PCN composites were synthesized by a facile in situ method that tightly combined PCN and MIO. The successful construction of MIO/PCN was determined by XRD, TEM, and XPS analysis. The best-performing sample was determined to be the 2.5 wt% MIO/PCN composite, which could degrade RhB almost 100% within 50 min under LED light irradiation. The reason for the excellent degradation capability of MIO/PCN was revealed by DRS, PL/TRPL, and EIS analysis. Experimental data showed the phase junction MIO provided a reliable electronic transmission channel for charge transfer and improved the separation efficiency of electron and hole. The active species were identified as ·O_2_^−^ and ·OH by photodegradation reaction containing scavenger and EPR. Additionally, the durability and stability of the MIO/PCN were upheld to an excellent degree after four cycle tests. Our work provides an avenue for the application of phase junction materials in photocatalysis.

## Data Availability

Not applicable.

## Supplementary Materials

The following supporting information can be downloaded at: https://www.mdpi.com/article/10.3390/ijms232214293/s1.

## References

[1] Zhang L., Song Y., Li Y., Yin Y., Cai Y. (2022). Role of light in methylmercury photodegradation: From irradiation to absorption in the presence of organic ligands. Sci. Total Environ..

[2] Smulek W., Bielan Z., Pacholak A., Zdarta A., Zgola-Grzeskowiak A., Zielinska-Jurek A., Kaczorek E. (2021). Nitrofurazone Removal from Water Enhanced by Coupling Photocatalysis and Biodegradation. Int. J. Mol. Sci..

[3] Liu X., Huang W.-Y., Zhou Q., Chen X.-R., Yang K., Li D., Dionysiou D.D. (2020). Ag-decorated 3D flower-like Bi_2_MoO_6_/rGO with boosted photocatalytic performance for removal of organic pollutants. Rare Met..

[4] Vinothkumar K., Jyothi M., Lavanya C., Sakar M., Valiyaveettil S., Balakrishna R.G. (2022). Strongly co-ordinated MOF-PSF matrix for selective adsorption, separation and photodegradation of dyes. Chem. Eng. J..

[5] Zeng R., Lian K., Su B., Lu L., Lin J., Tang D., Lin S., Wang X. (2021). Versatile Synthesis of Hollow Metal Sulfides via Reverse Cation Exchange Reactions for Photocatalytic CO_2_ Reduction. Angew. Chem. Int. Ed. Engl..

[6] Xu Y., Zhang Y., Wang X., Wang Z., Huang L., Wu H., Ren J., Gu C., Chen Z. (2022). Enhanced photodegradation of tylosin in the presence of natural montmorillonite: Synergistic effects of adsorption and surface hydroxyl radicals. Sci. Total Environ..

[7] Yu Z., Yang K., Yu C., Lu K., Huang W., Xu L., Zou L., Wang S., Chen Z., Hu J. (2022). Steering Unit Cell Dipole and Internal Electric Field by Highly Dispersed Er atoms Embedded into NiO for Efficient CO_2_ Photoreduction. Adv. Funct. Mater..

[8] Divakaran K., Baishnisha A., Balakumar V., Perumal K.N., Meenakshi C., Kannan R.S. (2021). Photocatalytic degradation of tetracycline under visible light using TiO_2_@sulfur doped carbon nitride nanocomposite synthesized via in-situ method. J. Environ. Chem. Eng..

[9] Zhang L., Zhang J., Xia Y., Xun M., Chen H., Liu X., Yin X. (2020). Metal-Free Carbon Quantum Dots Implant Graphitic Carbon Nitride: Enhanced Photocatalytic Dye Wastewater Purification with Simultaneous Hydrogen Production. Int. J. Mol. Sci..

[10] Li S., Cai M., Liu Y., Zhang J., Wang C., Zang S., Li Y., Zhang P., Li X. (2022). In-situ constructing C_3_N_5_ nanosheets/Bi_2_WO_6_ nanodots S-scheme heterojunction with enhanced structural defects for efficiently photocatalytic removal of tetracycline and Cr(VI). Inorg. Chem. Front..

[11] Lin L., Lin Z., Zhang J., Cai X., Wang X. (2020). Molecular-level insights on the reactive facet of carbon nitride single crystals photocatalysing overall water splitting. Nat. Catal..

[12] Pei Z., Gu J., Wang Y., Tang Z., Liu Z., Huang Y., Zhao J., Chen Z., Zhi C. (2017). Component matters: Paving the roadmap toward enhanced electrocatalytic performance of graphitic C_3_N_4_-based catalysts via atomic tuning. ACS Nano.

[13] Fang Y., Hou Y., Fu X., Wang X. (2022). Semiconducting Polymers for Oxygen Evolution Reaction under Light Illumination. Chem. Rev..

[14] Kumar A., Raizada P., Thakur V., Khan A.A.P., Singh N., Singh P. (2021). An overview on polymeric carbon nitride assisted photocatalytic CO_2_ reduction: Strategically manoeuvring solar to fuel conversion efficiency. Chem. Eng. Sci..

[15] Zhan H., Zhou Q., Li M., Zhou R., Mao Y., Wang P. (2022). Photocatalytic O_2_ activation and reactive oxygen species evolution by surface B-N bond for organic pollutants degradation. Appl. Catal. B Environ..

[16] Di G., Zhu Z., Zhang H., Zhu J., Qiu Y., Yin D., Kuppers S. (2019). Visible-light degradation of sulfonamides by Z-scheme ZnO/g-C_3_N_4_ heterojunctions with amorphous Fe_2_O_3_ as electron mediator. J. Colloid. Interface Sci..

[17] Wang X., Maeda K., Thomas A., Takanabe K., Antonietti M. (2009). A metal-free polymeric photocatalyst for hydrogen production from water under visible light. Nat. Mater..

[18] Gao S., Liu Y., Zhu J., Wang Y., Han X., Xia X., Zhao X. (2021). The synthesis of novel FeS_2_/g-C_3_N_4_ nanocomposites for the removal of tetracycline under visible-light irradiation. Environ. Sci. Water Res. Technol..

[19] Wei X., Wang X., Pu Y., Liu A., Chen C., Zou W., Zheng Y., Huang J., Zhang Y., Yang Y. (2021). Facile ball-milling synthesis of CeO_2_/g-C_3_N_4_ Z-scheme heterojunction for synergistic adsorption and photodegradation of methylene blue: Characteristics, kinetics, models, and mechanisms. Chem. Eng. J..

[20] Zhao H., Jian L., Gong M., Jing M., Li H., Mao Q., Lu T., Guo Y., Ji R., Chi W. (2022). Transition-Metal-Based Cocatalysts for Photocatalytic Water Splitting. Small Struct..

[21] Shao B., Wang J., Liu Z., Zeng G., Tang L., Liang Q., He Q., Wu T., Liu Y., Yuan X. (2020). Ti_3_C_2_T_x_ MXene decorated black phosphorus nanosheets with improved visible-light photocatalytic activity: Experimental and theoretical studies. J. Mater. Chem. A.

[22] Shahiduzzaman M., Visal S., Kuniyoshi M., Kaneko T., Umezu S., Katsumata T., Iwamori S., Kakihana M., Taima T., Isomura M. (2018). Low-temperature-processed brookite-based TiO_2_ heterophase junction enhances performance of planar perovskite solar cells. Nano Lett..

[23] Zhang J., Chen X., Bai Y., Li C., Gao Y., Li R., Li C. (2019). Boosting photocatalytic water splitting by tuning built-in electric field at phase junction. J. Mater. Chem. A.

[24] Gao Y., Zhu J., An H., Yan P., Huang B., Chen R., Fan F., Li C. (2017). Directly Probing Charge Separation at Interface of TiO_2_ Phase Junction. J. Phys. Chem. Lett..

[25] Zhang J., Xu Q., Feng Z., Li M., Li C. (2008). Importance of the relationship between surface phases and photocatalytic activity of TiO_2_. Angew. Chem..

[26] Ai Z., Zhao G., Zhong Y., Shao Y., Huang B., Wu Y., Hao X. (2018). Phase junction CdS: High efficient and stable photocatalyst for hydrogen generation. Appl. Catal. B Environ..

[27] Liu F., Shi R., Wang Z., Weng Y., Che C.M., Chen Y. (2019). Direct Z-Scheme Hetero-phase Junction of Black/Red Phosphorus for Photocatalytic Water Splitting. Angew. Chem. Int. Ed. Engl..

[28] Han L., Jing F., Luo X., Zhong Y.-L., Wang K., Zang S.-H., Teng D.-H., Liu Y., Chen J., Yang C. (2021). Environment friendly and remarkably efficient photocatalytic hydrogen evolution based on metal organic framework derived hexagonal/cubic In_2_O_3_ phase-junction. Appl. Catal. B Environ..

[29] Friedmann D., Caruso R. (2021). Indium Oxides and Related Indium-based Photocatalysts for Water Treatment: Materials Studied, Photocatalytic Performance, and Special Highlights. Sol. RRL.

[30] Feng Y., Yan T., Wu T., Zhang N., Yang Q., Sun M., Yan L., Du B., Wei Q. (2019). A label-free photoelectrochemical aptasensing platform base on plasmon Au coupling with MOF-derived In_2_O_3_@g-C_3_N_4_ nanoarchitectures for tetracycline detection. Sens. Actuators B Chem..

[31] Jin L.-N., Liu Q., Sun W.-Y. (2013). Size-controlled indium(iii)-benzenedicarboxylate hexagonal rods and their transformation to In_2_O_3_ hollow structures. Cryst. Eng. Comm..

[32] Wang S., Guan B.Y., Lou X.W.D. (2018). Construction of ZnIn_2_S_4_-In_2_O_3_ Hierarchical Tubular Heterostructures for Efficient CO_2_ Photoreduction. J. Am. Chem. Soc..

[33] Li R., Sun L., Zhan W., Li Y., Wang X., Han X. (2018). Engineering an effective noble-metal-free photocatalyst for hydrogen evolution: Hollow hexagonal porous micro-rods assembled from In_2_O_3_@carbon core-shell nanoparticles. J. Mater. Chem. A.

[34] Sun D., Wang W., Zhang N., Liu C., Li X., Zhou J., Ruan S. (2022). G-C_3_N_4_/In_2_O_3_ composite for effective formaldehyde detection. Sens. Actuators B Chem..

[35] Xu M., Zhao X., Jiang H., Chen S., Huo P. (2021). MOFs-derived C-In_2_O_3_/g-C_3_N_4_ heterojunction for enhanced photoreduction CO_2_. J. Environ. Chem. Eng..

[36] Jin X., Guan Q., Tian T., Li H., Han Y., Hao F., Cui Y., Li W., Zhu Y., Zhang Y. (2020). In_2_O_3_/boron doped g-C_3_N_4_ heterojunction catalysts with remarkably enhanced visible-light photocatalytic efficiencies. Appl. Surf. Sci..

[37] Uddin A., Rauf A., Wu T., Khan R., Yu Y., Tan L., Jiang F., Chen H. (2021). In_2_O_3_/oxygen doped g-C_3_N_4_ towards photocatalytic BPA degradation: Balance of oxygen between metal oxides and doped g-C_3_N_4_. J. Colloid. Interface Sci..

[38] Pan Z., Zhao M., Zhuzhang H., Zhang G., Anpo M., Wang X. (2021). Gradient Zn-Doped Poly Heptazine Imides Integrated with a van der Waals Homojunction Boosting Visible Light-Driven Water Oxidation Activities. ACS Catal..

[39] Vinoth S., Rajaitha P., Venkadesh A., Devi K.S.S., Radhakrishnan S., Pandikumar A. (2020). Nickel sulfide-incorporated sulfur-doped graphitic carbon nitride nanohybrid interface for non-enzymatic electrochemical sensing of glucose. Nanoscale Adv..

[40] Zang S., Cai X., Chen M., Teng D., Jing F., Leng Z., Zhou Y., Lin F. (2022). Tunable Carrier Transfer of Polymeric Carbon Nitride with Charge-Conducting CoV_2_O_6_·2H_2_O for Photocatalytic O_2_ Evolution. Nanomaterials.

[41] Cai M., Li R., Wang F., Guo X., Bai Q., Sun L., Han X. (2017). Architecture of designed hollow indium oxide microspheres assembled by porous nanosheets with high gas sensing capacity. J. Alloys Compd..

[42] Zhang K., Zhou M., Yang K., Yu C., Mu P., Yu Z., Lu K., Huang W., Dai W. (2022). Photocatalytic H_2_O_2_ production and removal of Cr (VI) via a novel Lu_3_NbO_7_: Yb, Ho/CQDs/AgInS_2_/In_2_S_3_ heterostructure with broad spectral response. J. Hazard. Mater..

[43] Sprynskyy M., Szczyglewska P., Wojtczak I., Nowak I., Witkowski A., Buszewski B., Feliczak-Guzik A. (2021). Diatom Biosilica Doped with Palladium(II) Chloride Nanoparticles as New Efficient Photocatalysts for Methyl Orange Degradation. Int. J. Mol. Sci..

[44] Chen M., Xu J., Tang R., Yuan S., Min Y., Xu Q., Shi P. (2022). Roles of microplastic-derived dissolved organic matter on the photodegradation of organic micropollutants. J. Hazard. Mater..

[45] Adeleke J., Theivasanthi T., Thiruppathi M., Akomolafe T., Alabi A. (2018). Photocatalytic degradation of methylene blue by ZnO/NiFe_2_O_4_ nanoparticles. Appl. Surf. Sci..

[46] Yadav R., Kumar R., Aliyan A., Dobal P.S., Biradar S., Vajtai R., Singh D.P., Martí A.A., Ajayan P.M. (2020). Facile synthesis of highly fluorescent free-standing films comprising graphitic carbon nitride (g-C_3_N_4_) nanolayers. New J. Chem..

[47] Li S., Cai M., Liu Y., Wang C., Yan R., Chen X. (2023). Constructing Cd_0.5_Zn_0.5_S/Bi_2_WO_6_ S-scheme heterojunction for boosted photocatalytic antibiotic oxidation and Cr (VI) reduction. Adv. Powder Mater..

[48] Fan G., Luo J., Guo L., Lin R., Zheng X., Snyder S.A. (2018). Doping Ag/AgCl in zeolitic imidazolate framework-8 (ZIF-8) to enhance the performance of photodegradation of methylene blue. Chemosphere.

[49] Lin B., Chen H., Zhou Y., Luo X., Tian D., Yan X., Duan R., Di J., Kang L., Zhou A. (2021). 2D/2D atomic double-layer WS_2_/Nb_2_O_5_ shell/core nanosheets with ultrafast interfacial charge transfer for boosting photocatalytic H_2_ evolution. Chin. Chem. Lett..

[50] Yan L., Gao H., Chen Y. (2021). Na-Doped Graphitic Carbon Nitride for Removal of Aqueous Contaminants via Adsorption and Photodegradation. ACS Appl. Nano Mater..

[51] Mu P., Zhou M., Yang K., Chen X., Yu Z., Lu K., Huang W., Yu C., Dai W. (2021). Cd_0.5_Zn_0.5_S/CoWO_4_ Nanohybrids with a Twinning Homojunction and an Interfacial S-Scheme Heterojunction for Efficient Visible-Light-Induced Photocatalytic CO_2_ Reduction. Inorg. Chem..

