# Supplementary material for: Rhombohedral/Cubic In2O3 Phase Junction Hybridized with Polymeric Carbon Nitride for Photodegradation of Organic Pollutants
Source: Int J Mol Sci. 2022 Nov 18;23(22):14293. doi: 10.3390/ijms232214293 (PMC9695553; doi:10.3390/ijms232214293)
Supplement: Supplementary file 1 [file ijms-23-14293-s001.zip › ijms-1972621-supplementary.pdf]

Rhombohedral corundum/cubic In_2O_3 phase-junction hybridized with Polymeric Carbon Nitride for Photodegradation of Organic Pollutants

Xiaorong Cai [1,+], Yaning Wang [1], Feng Lin [2,*], Shuting Tang [1], Liuye Mo [1], Yixian Zang [1], Zhe Leng [1,*], Fei Jing [1,+], Shaohong Zang [1,*]

Methods

1.1 Preparation of MIL-68(In)-NH_2

The precursor MIL-68(In)-NH_2 was prepared by a hydrothermal method. Typically, 1.3 mmol 2-aminoterephthalic acid and 3.8 mmol $In(NO_3)_3{\cdot}xH_2O$ were dissolved in 6.2 mL DMF, respectively. The solutions were mixed under continuously stirring. Then the mixture solution was transferred into an autoclave and reacted at 125 ˚C for 5 h in an oven. After the reaction ended, the resulting products were collected and washed with methanol several times. Finally, the collected MIL-68(In)-NH_2 sample was dried at 80 ˚C overnight.

1.2 Preparation of MIO/PCN and PCN

MIO/PCN samples were obtained by an in-situ method. Certain amounts of MIL-68(In)-NH_2 (5, 10, 20 mg) and 12 g urea were mixed in a glass bottle with a little bit of distilled water and stirred at 80 °C until the water evaporated completely. The obtained solid was transferred into a crucible and calcined at 500 ˚C with 2 ˚C min^{-1} in a muffle furnace. After reaction for 2 h, the products were collected and named *x wt*% MIO/PCN, where *x* means the calculated conversion rate of MIO.

Pure PCN was synthesized in the same condition without the addition of MIL-68(In)-NH_2.

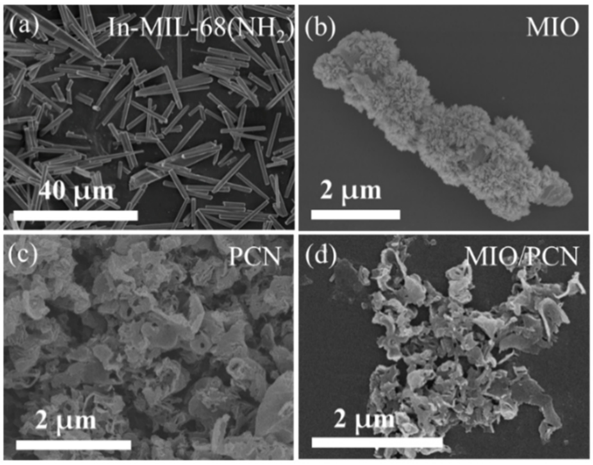

Figure S1. SEM images of (a) In-MIL-68(NH_2), (b) MIO, (c) PCN, (d) 2.5 *wt*% MIO/PCN samples.

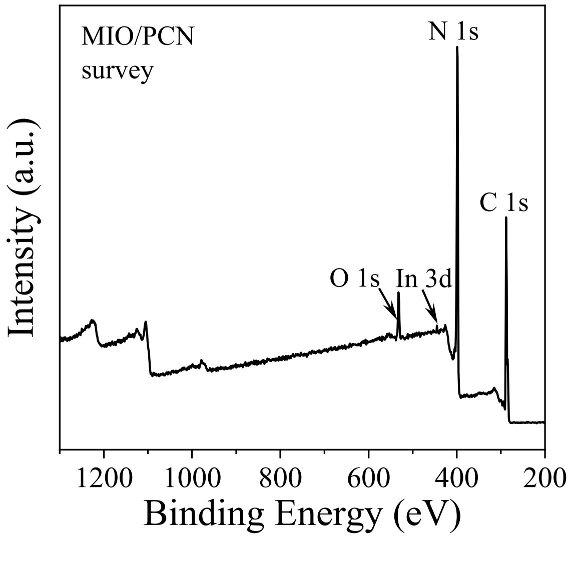

Figure S2. Survey XPS spectra of 2.5 *wt*% MIO/PCN samples.
